# Unraveling the Connection: Pancreatic Cancer Cells and Schwann Cells

**DOI:** 10.3390/jcm13061785

**Published:** 2024-03-20

**Authors:** Ingrid Garajová, Francesca Trentini, Francesco Leonardi, Elisa Giovannetti

**Affiliations:** 1 Medical Oncology Unit, University Hospital of Parma, 43100 Parma, Italy; francesca.trentini@unipr.it (F.T.); il-france@hotmail.com (F.L.); 2 Department of Medical Oncology, Lab of Medical Oncology, Cancer Center Amsterdam, Amsterdam UMC, VU University Medical Center (VUmc), 1007 MB Amsterdam, The Netherlands; elisa.giovannetti@gmail.com; 3 Cancer Pharmacology Lab, AIRC Start-Up Unit, 56017 Pisa, Italy; 4 Fondazione Pisana per la Scienza, 56017 San Giuliano Terme, Italy

**Keywords:** pancreatic ductal adenocarcinoma, neural invasion, Schwann cells, tumor microenvironment, cancer pain

## Abstract

Pancreatic ductal adenocarcinoma is one of the most lethal solid malignancies, characterized by its aggressiveness and metastatic potential, with a 5-year survival rate of only 13%. Progress in the management of metastatic disease has been modest. A robust connection between nervous system and tumor progression exists, with prominent neural alterations having been observed during pancreatic cancer’s progression, including neural hypertrophy, neural density, and neural remodeling. The pancreatic tumor microenvironment includes s set of cells and structures that constantly dialogue with cancer cells, influencing its growth and behavior. The microglia is key cellular components of the tumor microenvironment, and Schwann cells are the principal glial cells in the peripheral neural system. Schwann cells can regulate changes in the tumor microenvironment and immune responses by secreting a variety of factors and can support a tumor’s invasion of nerves and distant metastasis, with further pain exacerbation. Schwann cells secrete various pain-related molecules, such as the neural growth factor, to mediate the activation of primary sensory neurons, leading to pain induction. The binding of the neural growth factor to tropomyosin receptor kinase A is an important signaling mechanism for pain perception in humans. Consequently, directing efforts towards targeting neural invasion may provide an alternative strategy to improve the prognosis of and alleviate pain in patients with pancreatic cancer.

## 1. Neural Invasion in Pancreatic Cancer

Pancreatic ductal adenocarcinoma (PDAC) has the highest mortality rate of all major solid tumors and is currently the fourth leading cause of cancer-related deaths, with a 5-year overall survival (OS) rate of only 13% [1]. One of the pathological hallmarks of PDAC is neural invasion (NI). Among all solid tumors, NI has the highest prevalence in PDAC [2,3,4]. Interestingly, only about 25% of the early stages of PDAC do not present NI, suggesting that it could represent an early symptomatic phase of cancer progression. Cancer invasion damages the neural sheath and correlates with increased neural density and remodeling, resulting in both neuropathic and inflammatory pain. Moreover, the occurrence of NI has been correlated with poor outcomes after PDAC surgery. In a retrospective assessment by Crippa et al. involving 778 patients with PDAC who had undergone surgical intervention between 2009 and 2014, it was revealed that NI was detected in 87% of cases. Importantly, NI emerged as an independent risk factor significantly influencing disease-free survival (DFS) and overall survival (OS) (*p* < 0.0001), establishing its potential role as a prognostic indicator [5,6].

NI is defined as the presence of cancer cells along nerves and/or within the epineural, perineural, and endoneural spaces of the neuronal sheath, including cases in which the cells encircle at least 33% of the nerves [2]. This is believed to promote tumor progression by providing and creating a pathway for the spread of cancer cells [7]. Three distinct types of NI exist: epineural, perineural, and endoneural invasions. In epineural invasions, cancer cells directly touch the epineurium but do not penetrate it; in perineural invasions, cancer cells are within the perineural sheet; and, lastly, in endoneural invasions, cancer cells have invaded, through the perineurium, the endoneural sheet [2,4]. The type of NI holds clinical relevance; in particular, patients with endoneural invasions often report more intense pain compared to those with perineural invasions [4]. Understanding these distinctions in NI types becomes crucial in addressing the varying clinical presentations and pain management strategies for individuals affected by PDAC.

Several reasons might explain the high prevalence of NI in PDAC. The pancreas is a highly innervated organ: the innervation of the head of the pancreas depends on the celiac plexus, that of the uncinate process from the superior mesenteric plexus, while those of the body and tail are innervated from the splenic and celiac plexus [8]. However, the reasons for neural tropism extend beyond a mere anatomical position. It is widely recognized that tumor and nerve cells engage in communication, creating a reciprocal exchange which fosters a microenvironment conductive to tumor growth, establishing a detrimental and self-sustaining cycle. This intricate interplay between tumor cells and nerve cells underscores the complexity of neural tropism in the context of cancer progression [8,9]. Nerve-secreted substances can promote tumor invasion and growth, while tumor-secreted molecular mediators can induce the growth and extension of nerve axons toward tumor cells [9]. This reciprocal interaction potentiates tumor development and also impacts its microenvironment, as the secreted neurotransmitters and growth factors can also act on endothelial and immune cells, then contributing to tumor inflammation and neoangiogenesis [10]. Tumors can stimulate their own innervation via neurogenesis (increasing the number of neurons during tissue cancer damage) and neural reprogramming (differentiation of nerves into different varieties). Moreover, tumor growth induces neural sprouting towards the tumor, which, in turn, initiates cancer cell–nerve crosstalk, called axonogenesis [11]. Therefore, a more comprehensive understanding of the molecular mechanisms underlying NI could potentially unveil a key “Achilles’ heel” of PDAC. Such insights might pave the way for innovative treatment strategies aimed at enhancing both the outcomes and quality of life of patients with PDAC.

## 2. Schwann Cells’ Plasticity and Their Involvement in Cancer Neural Invasion

The tumor microenvironment (TME) includes sets of cells and structures that constantly dialogue with cancer cells, influencing a cancer’s growth and behavior [12]. The microglia are key cellular components of the TME. Schwann cells (SCs) are the principal glial cells of the peripheral neural system and exist in almost every anatomical part of the body. As the main support cells of the peripheral neural system, SCs constitute 90% of the endoneural space [12]. They regulate neural growth, survival, and repair by producing a variety of growth factors, such as the nerve growth factor (NGF), the glial cell line-derived neurotrophic factor (GDNF), and the brain-derived neurotrophic factor (BDNF) [13]. Preclinical studies have demonstrated that a co-culture of cancer cells with dorsal root ganglion extract revealed a phenomenon whereby SCs guided cancer cell migration to the nerve and promoted tumor invasion in a contact-dependent manner, which depended on the SCs expressing nerve cell adhesion molecule-1 (NCAM1) [14,15]. SCs are highly plastic cells that undergo cellular reprogramming during the response to nerve injuries, which makes them a pluripotent cell pool able to develop and regenerate the peripheral neural system [12] and, moreover, have immunomodulatory potential. SCs surround the axonal process of nerve fibers, forming the myelin sheath. Together with axons, they become part of the endoneurium layer, a richly vascularized fragile fluid matrix containing elastin and collagen [3,7]. Importantly, the presence of SCs has been observed in precancerous tissues of PDAC, which demonstrates their potential role in the early stages of tumor development [16].

## 3. Enrollment and Activation of Schwann Cells in PDAC

Demir et al. have previously demonstrated that the tumor hypoxic environment and the secretion of interleukin-6 (IL-6) from SCs is a trigger that induces a transition of SCs from a quiescent to an active state [17]. Interestingly, the initial activation of SCs suppresses the activity of the microglia and astrocytes in the spinal cord by inhibiting afferent pain fibers, resulting in a probable diagnostic delay related to this temporary state of analgesia [17]. After peripheral nerve injury, myelinated SCs dedifferentiate into “repair SCs” (rSCs), with an unmyelinated phenotype capable of guiding nerve regrowth [12,18,19]. SC reprogramming involves the upregulation of several genes and the activation of multiple transcriptional mechanisms [20]. Among the main players, c-Jun, mitogen-activated protein kinase (MAPK) pathways, and Sonic Hedgehog (Shh) and chromatin modifications control and regulate the repair program [20]. In fact, the non-myelinating Schwann cell profile has been correlated with a poor survival rate in patients with PDAC [20]. A remarkably elevated expression of the transcription factor c-JUN, a key factor in regulating the Schwann cell repair program, has also been associated with a worse prognosis for patients with PDAC [21,22]. Repair SCs overexpress several proteins, such as the glial fibrous acid protein (GFAP) and the p75 neurotrophin receptor (p75NTR). In addition, rSCs release more chemokines and neurotrophic factors [20,21,23]. Deborde et al. have demonstrated that a dedifferentiated subtype of Schwann cells associated with physiological nerve repair is activated in cancer [19]. Interestingly, only dedifferentiated SCs and not their differentiated counterparts are found to have direct contact with tumor cells. In this way, SCs intercalate between tumor cells, leading to perineural invasion, which might present a reservoir for tumor cells also after what is considered “radical” surgery [9]. This might explain the high rate of recurrence even after apparently radical surgeries for pancreatic cancer.

## 4. Schwann Cells, Tumor Cells, and Macrophages

SCs and tumor cells interact through direct binding to plasma membrane proteins and via secretory molecules [9]. Among the plasma membrane proteins, NCAM1 directly contacts tumor cells with SCs. Similarly, the myelin-associated glycoprotein (MAG) expressed on SCs binds pancreatic cells expressing the transmembranous mucin MUC1, creating an adhesive interaction between the cells [24]. NCAM1 activation induces a conformational change in SCs by making membrane protrusions appear that facilitate their dispersion among tumor cells, promoting NI [9]. Thus, intercalating between tumor cells, SCs promote the breakdown of tumor cells from clusters [19]. SCs also communicate with tumor cells though secretory proteins, including the L1 cell adhesion molecule (L1CAM) and transforming growth factor beta (TGF-b) [25]. Soluble L1CAM secreted by SCs attracts tumor cells and, through the STAT3 signaling pathway, induces the production of metalloproteinases MMP2 and MMP9, which facilitate the breakdown of the extracellular matrix (ECM) [25]. TGF-b is released by SCs and binds to tumor cells by activating the SMAD2/3 signaling pathway, promoting NI in preclinical models [26]. The expression and release of TGF-b have also been seen to increase in patients with PDAC [9]. Recently, it has been shown that the autophagy of SCs is induced by the secretion of NGF from PDAC cells, with the subsequent nerve axon growth mediating NI [27]. This makes NGF identifiable as a possible future target for blocking NI.

**Figure 1:**
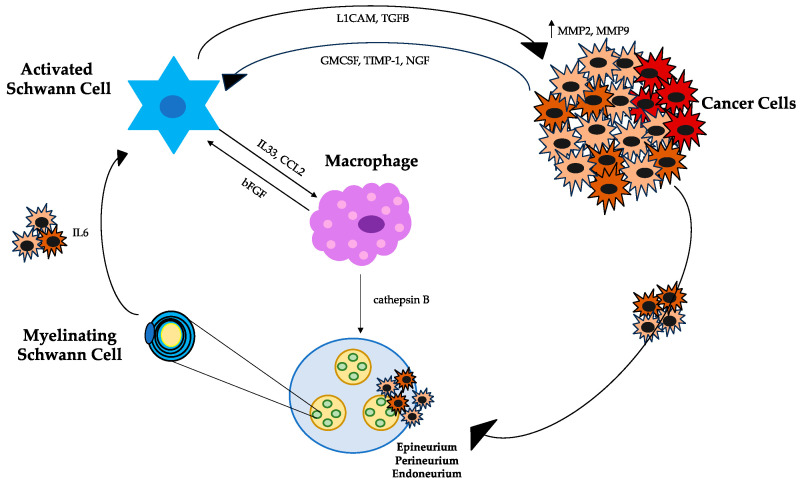
Interplay of signaling pathways: cellular dynamics within the pancreatic ductal adenocarcinoma microenvironment. The activation of SCs from a quiescent to an active state is induced by IL6 release from cancer cells. The hypoxic tumor state upregulates GM-CSF, promoting the migration and proliferation of SCs. Reciprocal paracrine signaling is established between cancer cells and SCs, with cancer cells releasing TIMP1 and NGF to influence SCs. Conversely, SCs release TGF-b and L1CAM, impacting cancer cells and contributing to NI. SCs play a multifaceted role by recruiting macrophages through the release of IL33 and CCL2. The CCL2-CCR2 pathway prompts TAMs to emit cathepsin B, thereby promoting nerve injury. TAMs, activated by IL33, release bFGF, establishing a positive feedback loop within the microenvironment. This intricate interplay highlights the dynamic and interconnected nature of cellular communication in the context of PDAC. Abbreviations: SCs, Schwann cells; GM-CSF, granulocyte–macrophage colony-stimulating factor; TIMP1, tissue inhibitor of metalloproteinases 1; NGF, nerve growth factor; TGF-b, transforming growth factor-beta; L1CAM, L1 cell adhesion molecule; NI, neural invasion; CCL2, chemokine ligand 2; TAMs, tumor-associated macrophages; bFGF, basic fibroblast growth factor; and PDAC, pancreatic ductal adenocarcinoma.

SCs–macrophages interaction is another example of communication within the TME that mediates NI processes [28,29]. SCs recruit macrophages through the release of IL33 and chemokine C-C motif chemokine ligand 2 (CCL2), which binds on the chemokine receptor 2 (CCR2) expressed on the surface of macrophages [9]. The CCL2-CCR2 pathway induces tumor-associated macrophages (TAMs) to release high levels of cathepsin B, which degrades collagen IV, a component of the perineurium, leading to nerve injury [9]. In this way, TAMs might promote tumor invasion by disrupting the perineurium [28,29]. Moreover, activated TAMs by IL33 release bFGF, which activates SCs via the PI3K-AKT-Myc signaling pathway, creating a positive feedback loop [28,29], as shown in Figure 1.

## 5. NGF/TrkA Signaling as a Therapeutic Target for the Treatment of Tumor Progression and Cancer Pain

**Figure 2:**
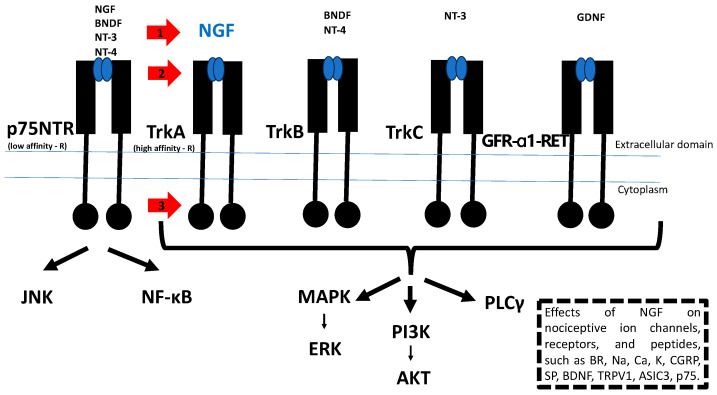
Pivotal role of the NGF/TrkA signaling pathway in PDAC. NGF binds to two receptors: low-affinity p75NTR and high-affinity TrkA receptors. NGF binding to a TrkA receptor mediates proliferation, differentiation, and survival via the activation of the Ras/MAPK, PI3K/AKT, and PLCγ pathways. NGF binding to the p75NTR receptor activates JNK and NF-κB. These mediate opposing effects of survival and apoptosis, respectively. Beyond NGF, several other neurotrophic molecules such as BDNF, NT-3, and NT-4/5 play a role in nerve growth. BDNF and NT-4 bind to the TrkB receptor and NT-3 to the TrkC receptor. GDNF binds to the GFR-α1-RET receptor. Effects of NGF increase the nociceptive ion channels, receptors, and peptides, such as BR, Na, Ca, K, CGRP, SP, BDNF, TRPV1, ASIC3, and p75. The red arrows show possible sites of NGF/TrkA signaling pathway inhibition. 1: NGF antagonists; 2: inhibitors of NGF binding to TrkA, and 3: TrkA inhibitors. Abbreviations: TrkA, tropomyosin receptor kinase A; NGF, nerve growth factor; MAPK, mitogen-activated protein kinase; PI3K, phosphatidylinositol-3-kinase; PLCγ, phospholipase Cγ; JNK, c-Jun N-terminal kinase; NF-κB, nuclear factor-κB; BDNF, brain-derived neurotrophic factor; neurotrophin-3 (NT-3); neurotrophin-4/5 (NT-4/5); TrkB, tropomyosin receptor kinase B; TrkC, tropomyosin receptor kinase C; GDNF, glial-derived neurotrophic factor; BR, bradykinin receptor; Na, sodium; Ca, calcium; K, potassium; CGRP, calcitonin gene-related peptide; SP, substance P; TRPV1, transient receptor potential cation channel subfamily V member 1; and ASIC3, acid-sensing ion channel 3.

SCs secrete several pain-related molecules, such as NGF, to mediate the activation of primary sensory neurons and induce pain [15]. There are two NGF receptors: one with a high affinity (tropomyosin receptor kinase (Trk) A) and the other with a low affinity for NGF (neurotrophin receptor (p75NTR) [30]. NGF binds to Trk A, with subsequent modulation of the function of nociceptor-expressed proteins such as neurotransmitters (e.g., SP and CGRP), channels and receptors (e.g., P2X3, TRPV1, and ASIC-3), and structural molecules (e.g., neurofilaments and p11), leading to an increase in nociceptive activity (7). Moreover, NGF is expressed by tumor cells, inflammatory cells, and immune cells [15,31,32]. The activation of NGF/TrkA signaling induces, therefore, both tumor progression and cancer pain, and either NGF or TrkA might be a therapeutic target against cancer [30,33]. Therefore, NGF/TrkA inhibitors are expected to be useful for both cancer pain and tumorigenesis. Beyond NGF, several other neurotrophic molecules such as the brain-derived neurotrophic factor (BDNF), neurotrophin-3 (NT-3), and neurotrophin-4/5 (NT-4/5) play a role in nerve growth [30], as illustrated in Figure 2. Indeed, the treatment of PDAC xenografts in nude mice with neurotrophin antibodies, including anti-NGF, anti-BDNF, anti-NT-3, and anti- NT-4/5, resulted in a significant inhibition of tumor growth [30]. The inhibition of the NGF/TrkA signaling pathway can be achieved through various approaches. This includes the direct antagonism of NGF, exemplified by monoclonal antibodies like tanezumab and fulranumab. Alternatively, inhibition can occur by impeding the binding of NGF and TrkA, as demonstrated by experimental compounds like MNAC13 and Ale0540. Another strategy involves hindering the enzymatic activity of TrkA, with small molecules such as larotrectinib and entrectinib. Lastly, inhibiting downstream signaling pathways, including the Ras/MAPK, PI3K/AKT, and PLCγ pathways, as well as JNK or NF-κB, is another avenue that has been explored [30,34,35,36], as illustrated in Figure 2. Recently, the efficacy and safety of tanezumab was investigated in patients with cancer pain predominantly due to bone metastasis who were receiving background opioid therapy. A phase III placebo-controlled study has demonstrated the potential of tanezumab in reducing the pain caused by bone metastases, though adjudicated intra-articular pathologic fractures were only observed among tanezumab-treated subjects. Further, as the study above did not establish the sustained efficacy of anti-NGF therapy beyond 8 weeks, future research should focus on assessing the durability of the therapeutic efficacy of this treatment over more extended periods [37]. The disruption of the NGF–TrkA interaction at the gene expression level presents another intriguing possibility. Lei et al. showed that the gold nanocluster-assisted delivery of the siRNA of NGF allows efficient NGF gene silencing. It downregulates NGF expression in Panc-1 cells and in pancreatic tumors and has been shown to effectively inhibit tumor progression in a xenograft model derived from patients with PDAC, without adverse effects [38].

## 6. Conclusions and Future Directions

PDAC sculpts its microenvironment in order to maximize cancer cell growth and metastatic potential. NI presents an early event in tumorigenesis, and some studies have demonstrated that neurogenesis and axonogenesis are present even in pre-neoplastic lesions and probably contribute to early cancer initiation [11]. Nerves in tumors can regulate various biological process, such as angiogenesis, lymphangiogenesis, immunity and inflammation, fibroblasts and the extracellular matrix, DNA repair, and oncogene activation [39]. In particular, pancreatic tumor cells and nerves can influence each other to provide a suitable microenvironment for tumor survival and growth in which molecular, cellular, and metabolic mechanisms are all involved. Nerve-secreted substances can promote tumor invasion and growth, while tumor-secreted molecular mediators can induce the growth and extension of nerve axons toward tumor cells [10]. Such a dynamic communication between the nerves and PDAC cells further facilitates immune escape, tumor growth, and metastasis. SCs are believed to possess the potential to drive tumorigenesis, produce signals to promote cancer invasion, and reshape the ECM [40]. From a clinical perspective, the development of novel therapies targeting the modification of SCs’ repair phenotype holds promise for mitigating perineural invasion in PDAC. This approach not only has the potential to enhance patient outcomes but could also significantly improve the overall quality of life of individuals diagnosed with PDAC. Presently, anti-NGF therapy is predominantly utilized for chronic pain management in rheumatology and diabetic neuropathy settings. However, it is crucial to note that there is currently insufficient conclusive evidence regarding the efficacy of this approach in the oncological context. Further research and clinical studies are warranted to establish the effectiveness of anti-NGF therapy specifically in the realm of cancer treatment.

## Data Availability

The data presented in this study are available on request from the corresponding author.
